# 3D-Printed Poly(3-hydroxybutyrate-*co*-3-hydroxyhexanoate)-Cellulose-Based Scaffolds for Biomedical Applications

**DOI:** 10.1021/acs.biomac.3c00263

**Published:** 2023-08-17

**Authors:** Alberto Giubilini, Massimo Messori, Federica Bondioli, Paolo Minetola, Luca Iuliano, Gustav Nyström, Katharina Maniura-Weber, Markus Rottmar, Gilberto Siqueira

**Affiliations:** † Department of Management and Production Engineering (DIGEP), Politecnico di Torino, Torino 10129, Italy; ‡ Integrated Additive Manufacturing Centre (IAM@PoliTO), Politecnico di Torino, Torino 10129, Italy; § Department of Applied Science and Technology (DISAT), Politecnico di Torino, Torino 10129, Italy; ∥ Cellulose & Wood Materials Laboratory, Swiss Federal Laboratories for Materials Science and Technology (Empa), Dübendorf 8600, Switzerland; ⊥ Department of Health Sciences and Technology, ETH Zürich, Zürich 8092, Switzerland; # Biointerfaces, Swiss Federal Laboratories for Materials Science and Technology (Empa), St. Gallen 9014, Switzerland

## Abstract

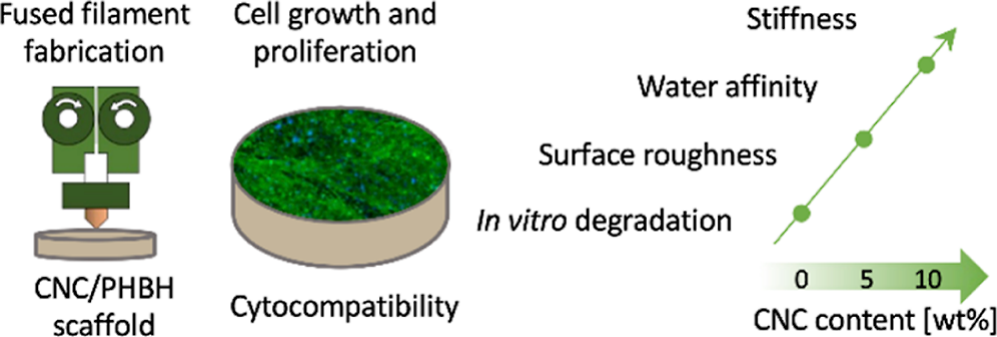

While biomaterials have become indispensable for a wide range of tissue repair strategies, second removal procedures oftentimes needed in the case of non-bio-based and non-bioresorbable scaffolds are associated with significant drawbacks not only for the patient, including the risk of infection, impaired healing, or tissue damage, but also for the healthcare system in terms of cost and resources. New biopolymers are increasingly being investigated in the field of tissue regeneration, but their widespread use is still hampered by limitations regarding mechanical, biological, and functional performance when compared to traditional materials. Therefore, a common strategy to tune and broaden the final properties of biopolymers is through the effect of different reinforcing agents. This research work focused on the fabrication and characterization of a bio-based and bioresorbable composite material obtained by compounding a poly(3-hydroxybutyrate-*co*-3-hydroxyhexanoate) (PHBH) matrix with acetylated cellulose nanocrystals (CNCs). The developed biocomposite was further processed to obtain three-dimensional scaffolds by additive manufacturing (AM). The 3D printability of the PHBH–CNC biocomposites was demonstrated by realizing different scaffold geometries, and the results of in vitro cell viability studies provided a clear indication of the cytocompatibility of the biocomposites. Moreover, the CNC content proved to be an important parameter in tuning the different functional properties of the scaffolds. It was demonstrated that the water affinity, surface roughness, and in vitro degradability rate of biocomposites increase with increasing CNC content. Therefore, this tailoring effect of CNC can expand the potential field of use of the PHBH biopolymer, making it an attractive candidate for a variety of tissue engineering applications.

## 1 Introduction

Some of the most significant challenges in modern biomedical research involve regenerative medicine, which is aimed at stimulating the repair of viable tissues after injury or damage through the use of biomaterials and biological growth factors.^1^ There is an increasing demand for soft and hard tissue engineered solutions to treat injuries, and such strategies should not only facilitate cell growth and new tissue formation but also depending on the type and location of the defect, they also need to withstand significant external loads applied to the injured area. Scaffolds for TE applications have been developed during the last decade for preclinical and clinical applications with traditional and well-known materials, e.g., metals,^2,3^ ceramics,^4,5^ polymers,^6,7^ or polymer/ceramic composites.^8,9^ For all scaffold materials, cytocompatibility is essential in order to avoid an adverse immune response and the risk of rejection. Besides this and depending on the application, the resorbability of an implantable scaffold is oftentimes desired so that the regeneration process and scaffold degradation can proceed jointly, leaving non-toxic degradation products and thus avoiding an additional procedure of surgical removal of the implant.^10^

For applications in tissue engineering and regeneration, polycaprolactone (PCL) and poly(lactic acid) (PLA) have found widespread use due to their well-known cytocompatibility and biodegradation. PCL was studied for applications in meniscus regeneration,^11^ but because of its limited mechanical performances, PCL is not a valid option for hard tissue replacement, such as cortical bones. PLA has superior mechanical properties compared to PCL, and previous research demonstrated the possibility to 3D print PLA scaffolds for trabecular bone replacement.^12,13^ However, the use of PLA for 3D printing is limited by a narrow processing window, a high melting temperature, its brittleness, and the risk of structure instability by chain hydrolysis.^14^

An emerging class of polymers suitable for a range of biomedical applications is the family of polyhydroxyalkanoates (PHAs),^15−17^ which are aliphatic biodegradable polyesters synthesized in the form of intracellular granules by a wide range of microorganisms. PHAs have attracted increasing attention for tissue engineering applications due to their bio-origin and biodegradability, as well as their cytocompatibility and resorbability.^18,19^ Depending on the biosynthetic production pathway, there is a wide variety of chemical PHA structures that vary in their monomeric composition or backbone chain-length, thereby offering a wide range of thermal and mechanical properties.^20^ To enhance the final properties of these biopolymers, PHAs have been compounded with organic^21,22^ or inorganic fillers,^23,24^ stabilizers, or anti-hydrolysis agents. For example, in our previous work, we used cellulose nanocrystals (CNCs) to improve the mechanical performance as well as the thermal stability of a PHA matrix.^25^ CNCs are attractive components due to their bio-origin, high aspect ratio, stiffness, cytocompatibility, and high versatility in their application fields.^26^ While some studies have already investigated the use of CNCs for biomedical aims and reported an enhancement in wettability and cellular adhesion for CNC–gelatin scaffolds^27^ or CNC aerogels,^28^ there is still plenty of room for in-depth investigation into the potential of PHA–CNC composites to produce stiffer scaffolds than the aforementioned aerogels.

Along with the ever-growing diffusion of PHAs, processing technologies have also been studied and discussed, and reports on PHA scaffold fabrication predominantly rely on traditional fabrication techniques such as solvent casting, salt leaching, or thermally induced phase separation (TIPS), which offer no control over final geometry or stress formation during the drying process, limited design freedom, no possibility of customization, and three-dimensional development.^29^ However, these limitations can be easily overcome by additive manufacturing (AM) techniques, which have found incredible success in various fields of application, including biomedical ones, due to the high degree of design freedom and the extraordinary level of customization of the final geometries^30−32^ that can even meet the architectural requirements of the defect site in patients.^33^ Many different AM approaches have been developed so far, but for polymer-based materials, extrusion-based technologies are the most widely applied and widely available. Among these techniques, fused filament fabrication (FFF) is especially popular thanks to its straightforwardness. It consists in heating up a continuous filament of a thermoplastic polymer above its melting temperature (*T*_m_) and extruding the material through a nozzle on a printing bed in a layer-by-layer approach. Due to instant cooling and hardening of the printed filament, determination of the optimal extrusion conditions of the melted filament is of major importance to ensure adhesion between the different layers.^34^ A valuable reason for the use of FFF for biomedical scaffold fabrication is the possibility to tune the porosity of the final scaffold to optimize different processes that are key for tissue function, such as cell migration, diffusion of oxygen and nutrients, waste product removal, and extracellular matrix production.^35^ This tunable macroporosity can also be useful to control the mechanical performance of the final construct, e.g., Young’s modulus,^36^ thus reproducing the properties of the target tissue as closely as possible and mitigating an important implantation problem such as stress concentrations on the injured area.^37^ Although several thermoplastic polymers can be processed by FFF, there are still limitations in the properties of commercially available filaments for biomedical applications. To be processed in FFF, the polymer should have sufficient thermal stability and an adequate rheological behavior to avoid a drop in molecular weight due to thermal degradation during the printing process.^38^ In this regard, polymer blending and filler compounding of the filament material have been described to lower the melting temperature, broaden the thermal stability range, or tailor the rheological properties, leading to a higher resolution of 3D-printed scaffolds.^39,40^

**Figure 1:**
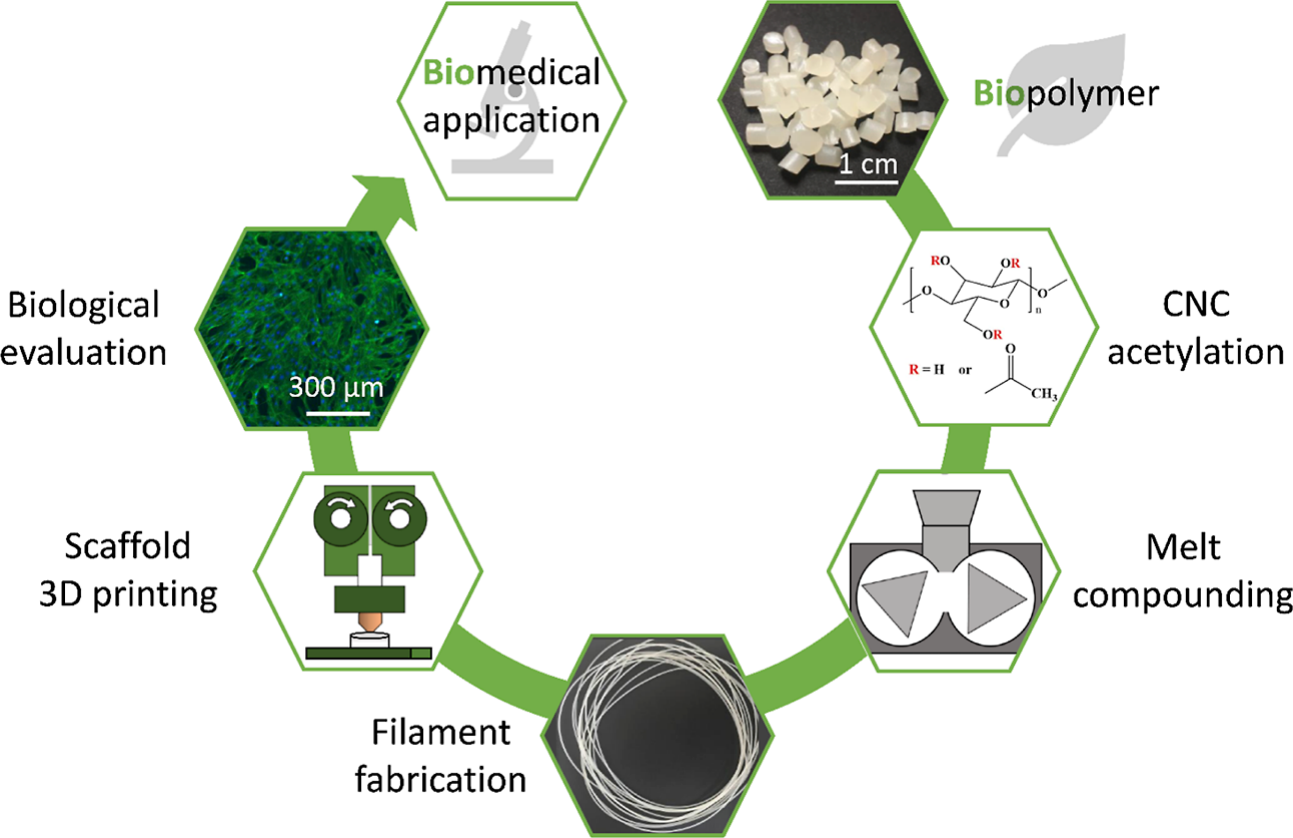
Schematic representation of the research workflow starting from the bio-sourced materials, the technological transformation, and the final characterization of the biomedical scaffolds.

Therefore, in the present study, we aimed to advance and support the use of a solvent-free, low-cost, and straightforward AM technology, such as FFF, for the fabrication of 3D-printed scaffolds. We wanted these scaffolds to be able to support tissue regeneration processes and to be bioresorbable so that they could be naturally reabsorbed by the human body at the end of the healing process. We also aimed to investigate the in vitro cytocompatibility of CNCs within a PHA matrix and further tune the water affinity, the surface roughness, and the in vitro degradation rate. A graphical representation of the structure of this research work is shown in Figure 1, where bio-sourced materials, PHA and CNCs, were first technologically processed via melt compounding and extrusion, to be later 3D printed as scaffolds for cell studies. The results of the characterization carried out suggest that the developed biocomposite has potential applicability for tissue regeneration and deserves to be further investigated. To the authors’ knowledge, studies on the development of fully bio-based materials for biomedical applications are still extremely limited. Here, the development, processing, and performance of a new PHBH–CNC biocomposite for tissue engineering applications are shown, which succeeds in combining the advantages of eco-friendly and renewable materials with the production of customizable designs by AM, which is particularly valuable in the biomedical field.

## 2 Materials and Methods

### 2.1 Materials

Poly(3-hydroxybutyrate-*co*-3-hydroxyhexanoate) (PHBH) containing 11 mol % of hydroxyhexanoate was supplied by MAIP Group (MAIP srl, Turin, Italy), with commercial code B6H N15, in pellet form. Before processing, PHBH pellets were dried in an oven at 85 °C overnight. CNCs with an average length (*L*) of 120 nm, an average diameter (*d*) of 6.5 nm, and an aspect ratio of ∼18 were purchased from CelluForce (CelluForce, Montréal, Québec, Canada). Acetic anhydride and sulfuric acid (95–97%) were purchased from Merck and used as received without further purification. Normal human dermal fibroblasts (NHDFs) were purchased from Promocell (NHDF-c adult, Lot no. 410Z037.5).

### 2.2 Methods

#### 2.2.1 Fabrication of Biocomposites

The preparation of acetylated CNCs and PHBH-based composites was performed as described in our previous work.^25^ Briefly, acetic anhydride (75 mL) was mixed with sulfuric acid (150 μL) used as a catalyst, and then CNCs (15 g) were added to this solution under mechanical stirring at 250 rpm for 8 h at 30 °C. Acetylated CNCs were then washed by centrifugation and redispersion four times with ethanol. The reaction time was chosen in accordance with the results presented in previous research, where 8 h of acetylation showed a fair balance between a sufficiently high degree of acetylation, evaluated by the absorption ratio between two peaks of the grafted acetyl group and the cellulose molecular skeleton, without compromising either the nanostructure or the morphology of the nanocrystals. The melt compounding between PHBH and acetylated CNCs was performed using a high-shear mixing Rheomix 300 (PolyLab OS, Thermo Electron Corporation, Germany) with roller rotors at a processing temperature of 145 °C, a mixing speed of 10 rpm, and a mixing time of 10 min. Final biocomposites were obtained at 0, 5, and 10 wt % filler contents. Hereafter, the label “PHBH” was used for neat PHBH, technologically processed as all other composites, and “PHBH_CNC_*XX*” for biocomposites, where *XX* represents the CNC content expressed as weight percent (5 and 10 wt %). For the FFF process, filaments were extruded at 145 °C through a die orifice with a diameter of 1.8 mm using a piston extruder (Rosand RH7 Flowmaster, Netzsch GmbH, Germany) at an extrusion speed of 8.5 mm min^–1^. Subsequently, the filaments were stored in a desiccator to prevent the absorption of environmental moisture, which would affect the 3D printing process.

#### 2.2.2 3D Printing of Scaffolds

The structure of the 3D-printed PHBH_CNC composite scaffolds was modeled using SolidWorks 2016 software (SolidWorks Corporation, USA). Specifically, a plane geometry, as shown in Figure 3a, was prepared for in vitro cytocompatibility tests, and a grid-filled geometry, as shown in Figure 3e–g, was prepared to investigate the 3D printability of fully interconnected structures with a 90° orientation step between each layer. The best printing conditions for this type of composite (CNC-PHBH), previously found in our work,^25^ were used. All geometries were printed with a nozzle temperature of 180 °C, a platform temperature of 50 °C, and a printing speed of 5 mm s^–1^ on a commercial FFF-based 3D printer (TREAStilla 3D FFF, Stilla3D, Italy). The nozzle diameter and the layer height were 0.4 and 0.32 mm, respectively. The filling density was set to 100% for plane geometry and to 60% for grid-filled geometry.

### 2.3 Characterization

#### 2.3.1 Tensile Testing

To measure the Young’s modulus (*E*), the ultimate tensile strength (UTS) and the elongation at break (ε_b_) of the biocomposites, hot-pressed films were fabricated at 120 °C for 30 s and cut out in samples with dimensions of 40 × 3 × 0.3 mm^3^ (length × width × thickness) to be further analyzed via tensile testing. To perform this characterization, a stress/strain test was carried out on a DMA Q800 (TA Instruments, New Castle, Delaware, USA) machine equipped with a film tension clamp. The tests were performed in controlled force mode, a preload force of 0.5 N along with a ramp force of 0.1 N min^–1^ was chosen, and five specimens were tested for each composition.

#### 2.3.2 Optical Microscopy

To investigate the shape fidelity and evaluate the overall quality of FFF 3D-printed samples, the grid-filled geometry was examined using an Axioplan microscope from Zeiss (Zeiss, Oberkochen, Germany) equipped with cross-polarized filters.

#### 2.3.3 Scanning Electron Microscopy (SEM)

The morphological evaluation of the scaffold disintegration process in a simulated body environment was studied using a Phenom XL G2 Desktop scanning electron microscope (Thermo Fisher Scientific, Waltham, Massachusetts, USA) at an accelerating voltage of 15 kV. Each sample was mounted on carbon tape and sputter-coated with a layer of gold for 3 min at 10^–3^ mbar and 10 mA current flow (SPI Supplies, Complete Sputter Coating System, West Chester, Pennsylvania, USA).

#### 2.3.4 X-ray Computed Tomography (X-CT)

A micro-CT scan model Phoenix v|tome|x S240 (GE Baker Hughes-Waygate Technologies, Wunstorf, Germany) was used to inspect the 3D-printed samples. The same scanning parameters were used for each scan: voxel size of 14.8 μm, voltage at 180 kV, current at 85 μA, timing of 100 ms, and 1500 images were acquired. The reconstruction of the X-ray images into a 3D model was performed with datos|reconstruction software, and VG Studio Max software (version 3.4) by Volume Graphics (Hexagon Metrology-Volume Graphics, Heidelberg, Germany) was used for visualization and analysis. Before starting the dimensional analysis, a surface determination was conducted with the *Isovalue-(based)* approach. Based on surface determination results, the *nominal/actual comparison* analysis module of VG Studio software was selected for the evaluation of printing accuracy.

#### 2.3.5 Atomic Force Microscopy

Atomic force microscopy (AFM) images (10 × 10 μm^2^) were obtained with an ICON3 AFM (Bruker, USA) in tapping mode with Bruker silicon tips (RETESPA-300). 3D-printed samples were attached to a microscopy glass slide with a hard carbon double tape, cleaned with N_2_ flow, and stored under vacuum in a desiccator to avoid contamination prior to the experiments. The images were processed with NanoScope Analysis from Bruker to obtain measurements of the root mean square (RMS) surface roughness.

#### 2.3.6 Water Absorption

The water affinity of biocomposites was evaluated through the determination of their water absorption degree according to the ISO 62:2008 standard with distilled water at 23 ± 1 °C for an immersion time of up to 16 days. The sample size was 75 × 25 × 0.5 mm^3^ and, before the water immersion, all samples were oven dried at 50 °C until a constant mass was reached.

At each experiment time point, samples were taken from the immersion bath, and the surface water was removed with filter paper, weighed to the nearest 0.1 mg, and then submerged back in the water bath. All tests were carried out in triplicate.

The water uptake *M*_t_ was calculated by the following equation
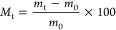
where *m*_t_ represents the sample mass after an immersion time *t* and *m*_0_ is the initial mass of the dry sample before immersion.

The saturation time is considered the time after which the water uptake profiles get to an asymptotic value and no additional weight gain is observed. Therefore, the saturation mass *M*_s_ was taken as the *M*_t_ at equilibrium.

The water diffusion coefficient *D* of biocomposites was calculated by determining the solution to Fick’s first law, which, specifically for plane sheet geometry of thickness *d*, can be simplified using Stefan’s approximation^41^
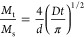
where the water uptake at time *t* is expressed as *M*_t_ and the water uptake at equilibrium as *M*_s_.

This estimation is used for describing the earlier stages of water uptake, usually for *M*_t_/*M*_s_ ≤ 0.5. By plotting the *M*_t_/*M*_s_ ratios as a function of the square root of time, the slope of the plot θ can be calculated,^42^ and consequently, *D* can be determined as follows
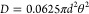


#### 2.3.7 Static Water Contact Angle

The surface wettability of all scaffolds, 3D printed via FFF, was investigated by determining the static water contact angle with an OCA20 contact angle system (DataPhysics, Germany) at 25 °C. About 6 μL was used as the volume for the water drop, and four different points of each sample were measured to calculate the average value and the standard deviation.

#### 2.3.8 In Vitro Degradation

The degradation experiments were carried out according to ISO 10993-13:2010. The 3D-printed scaffold samples (10 × 10 × 0.5 mm^3^) were incubated (1 g: 30 mL) at 37 °C in an oxidizing environment, with an aqueous solution of H_2_O_2_ (10%) and 1.31 mM of CaCl_2_, as reported in previous research.^43^ The peroxide solution was changed every week to maintain a constant degradation environment. The specimens were dried until reaching weight constancy before and after degradation assays to determine weight loss (*W*_t_), expressed as
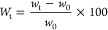
where *w*_t_ represents the sample weight after an immersion time *t* and *w*_0_ is the initial weight of the dry sample before immersion. Three test samples were used for each composition.

#### 2.3.9 Cellular Attachment and Proliferation

To investigate cell adhesion and proliferation on FFF 3D-printed scaffolds, samples were sterilized in 70% ethanol for 30 min and treated with air plasma for 30 s (PDC-32G Harrick Plasma). NHDFs were seeded on top of the samples at a density of 20,000 cells/cm^2^. On days 1 and 3, cells were fixed in a 10% formalin solution for 20 min, followed by 10 min of incubation in a 0.1% Triton-X solution to permeabilize the cell membrane. Thereafter, cells were stained for their cytoskeleton (Alexa Fluor 488-labeled phalloidin (1:200) A12379, Invitrogen) and for nuclei [4′,6-diamidino-2-phenylindole (DAPI) (1:1000), Sigma D9542]. Images were acquired with a confocal laser scanning microscope (LSM780, Zeiss).

## 3 Results and Discussion

### 3.1 Tensile Testing

**Figure 2:**
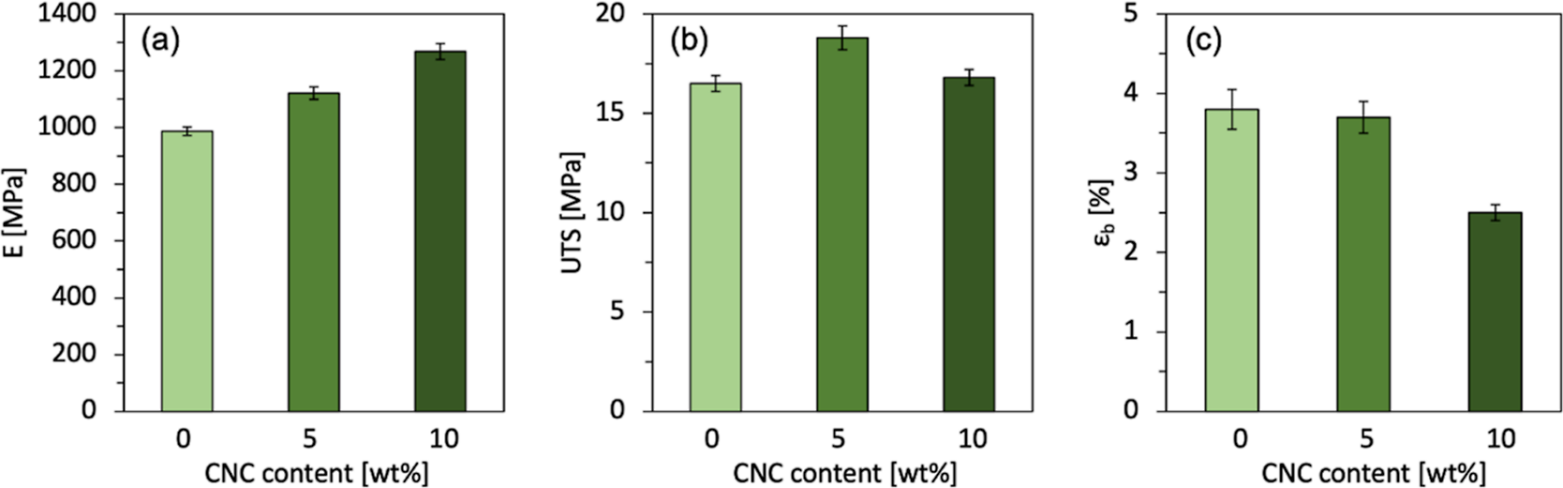
Mechanical properties of composites with increasing amounts of acetylated CNC content (0, 5, and 10 wt %): (a) Young’s modulus (*E*); (b) UTS; and (c) elongation at break (ε_b_).

The mechanical properties of the biocomposites were investigated via tensile testing of hot-pressed films, and the results for the different compositions are graphically illustrated in Figure 2. With the increasing CNC content, we can observe an increase in the Young’s modulus values of composites (Figure 2a), up to a maximum increase of almost 30%. Reasonably, this stiffer behavior occurs with a decrease in flexibility and hence a reduction of elongation at break (Figure 2c). This is most notable for the 10 wt % CNC concentration, where ε_b_ decreased from 3.8 to 2.5% compared to neat PHBH, which corresponds to a reduction of almost 35%. Importantly, however, no drop in tensile stress at break was encountered after compounding, and for a 5 wt % composition, a slight improvement can be observed (Figure 2b). The maintenance of UTS performance could be influenced by the higher rigidity of CNCs with respect to neat PHBH, which can be an indirect indication of the good affinity and adhesion between the functionalized CNCs and the biopolymer matrix, leading to an improved stress transfer mechanism at their interface. These results are consistent with a previous report by Li et al., who found the greatest enhancement of UTS with a 7 wt % concentration of acetylated CNCs in the PHBH matrix, above which a slight decrease could be noticed.^44^

### 3.2 Scaffold Morphology

**Figure 3:**
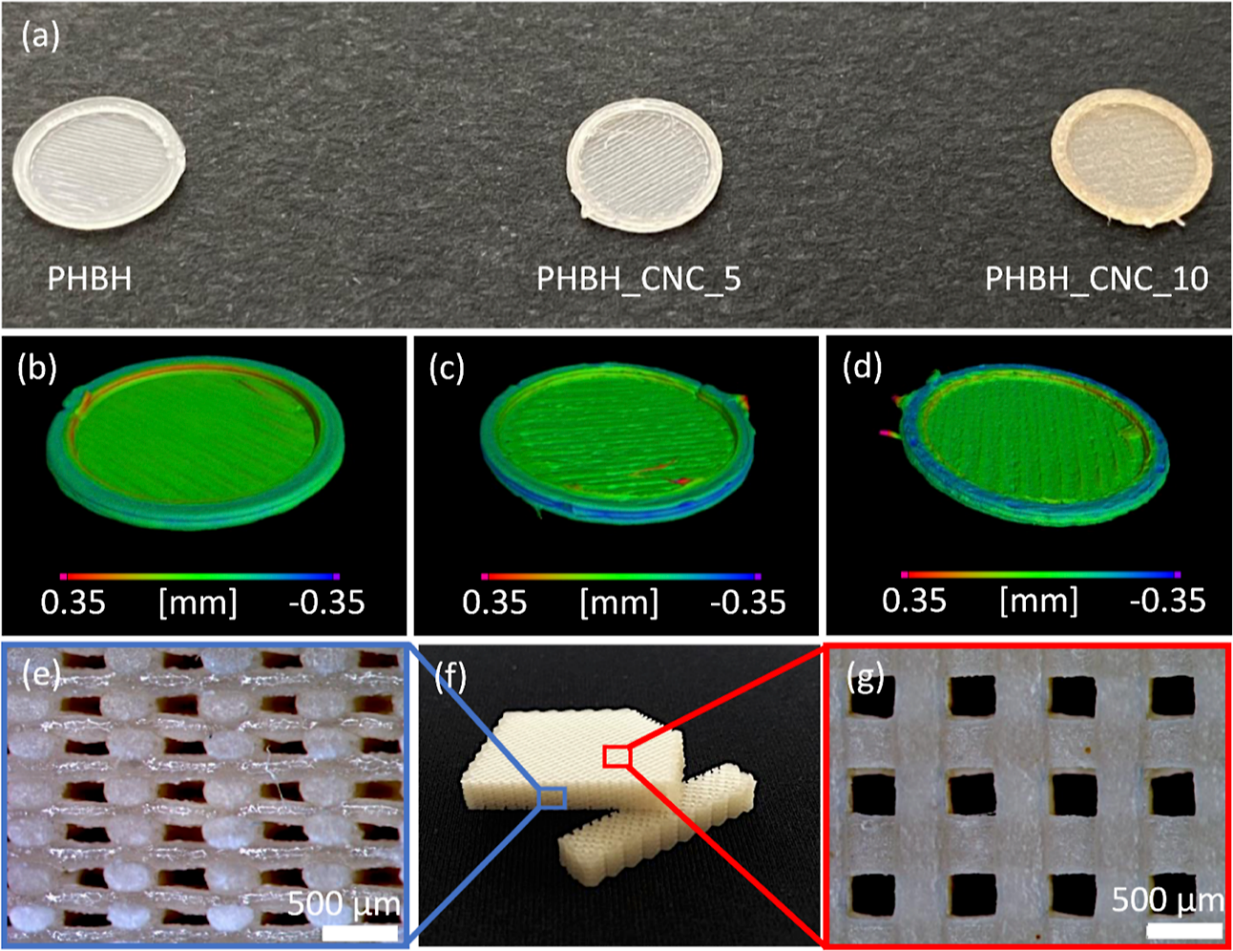
FFF 3D-printed biocomposites. (a) Illustration of 3D printed 100% infill geometries for cytocompatibility tests, fabricated with varied filaments: neat PHBH, PHBH_CNC_5, and PHBH_CNC_10. Evaluation of dimensional accuracy by the chromatic representation of deviation between actual and nominal dimensions of the different samples: (b) PHBH, (c) PHBH_CNC_5, and (d) PHBH_CNC_10. (f) Visual appearance and its associated representative (e) cross-section and (g) top views of scaffolds 3D printed by a filament of PHBH_CNC_5, with an alternation of 0–90° for directions of layers.

To assess the printability of CNC-based composites in 3D (*x*, *y*, and *z*), we fabricated by FFF both simple shapes, such as a plane geometry (Figure 3a) to evaluate cell attachment and proliferation, and scaffolds with a grid-filled geometry (Figure 3e–g). The sample structure, the definition of each extruded filament, and the pore size of the 3D-printed samples were investigated by visual and microscopy inspection.

The 3D printing accuracy of the specimens was evaluated by conducting X-CT scans of three different samples, one for each composition, and then they were first aligned to the original CAD model using the best-fit registration function on VG Studio software. After the alignment, the deviations between the actual 3D-printed geometries and the nominal ones of the CAD model were computed. A range of ±0.35 mm was chosen for all samples as a reference search distance to get comparable results for the dimensional deviation on the piece surface. The results of data comparisons and their deviations are illustrated in Figure 3b–d, where the photograph (Figure 3a) of the real pieces is compared to the colored deviation maps (Figure 3b–d). All three samples displayed good dimensional accuracy; for instance, upon expressing the deviation value within which 95% of the surface area falls as an indicative parameter, values of 0.15, 0.21, and 0.20 mm, respectively, are observed for the PHBH, PHBH_CNC_5, and PHBH_CNC_10 specimens. The biocomposites, compared to the neat biopolymer, exhibited slightly higher deviations also due to some 3D-printing defects, such as filaments’ squeezing during material deposition or minor smears.

Although several previous studies investigated the optimal size for the macroporosity of a scaffold, it was not possible to establish an unambiguous and certain value for tissue regeneration but rather a range of values identified according to the injured tissue. For example, the typical size of osteoblasts is around 20–50 μm,^45^ but pores larger than this have been reported to promote postimplant bone formation. Lim et al. reported that 90 μm pores better supported cell infiltration and vascularization compared to 30 μm pores but that the addition of 300 μm channels in the latter scaffolds could achieve similar results as with the larger pore sizes.^46^ Murphy et al., on the other hand, showed enhanced infiltration and osteoblast attachment, with pores of 325 μm when compared to pore sizes of 190 μm and lower.^47^ In line with this, Cheng et al. compared magnesium scaffolds with 250 and 400 μm pore sizes and showed that larger pore sizes led to enhanced vascularization and higher bone regeneration.^48^

In light of these reports, the internal macroporosity of 3D scaffolds was chosen to be approximately 300 μm. The porous structure of all samples was observed by SEM, and the mean pore size was calculated over ten measurements for each composition. Specifically, the values were 315 ± 16, 323 ± 26, and 317 ± 18 μm for PHBH, PHBH_CNC_5, and PHBH_CNC_10, respectively. Apparently, there is no direct correlation between the CNC content and control over scaffold pore size. As a matter of fact, for all compositions, it was always possible to recreate a well-defined and controlled porous structure with an average pore size of about 300 μm. Being representative for all compositions, 3D-printed PHBH_CNC_5 scaffolds with an infill density of 60% and an alternation of 90° for each layer are shown in Figure 3e–g. From their top and cross-sectional views, all scaffolds denote maintenance of the filamentary shape, a suitable printing quality, an appropriate self-supporting structure, which is composed of 20 layers with no collapsing parts or delaminated layers, and the porous structure reflects the original filling pattern, alternating each layer of 0–90°. The set layer height of 0.32 mm, which is 80% of the 3D printer nozzle diameter, i.e., 0.4 mm, guarantees an overlap between one layer and the following one. This overlay leads to the melt diffusion of the polymer chains between two adjacent layers and thus to enhanced interlayer adhesion, which can prevent delamination. This is particularly clear and visible from Figure 3e, where the height of each extruded layer is smaller compared to its width.

Most of the studies conducted so far in the field of biomedical application of polymer-CNC composites have been carried out with techniques such as solvent casting, freeze drying, hydrogels, or aerogel formation, which, however, do not include the possibility of having structure and macroporosity control. Here, adequate porosity control of the 3D structure was possible through the application of an AM approach. Comparing our data with those of previous studies that fabricated PHBH-based scaffolds with traditional techniques, they obtained much smaller pore sizes, as in the case of electrospinning, i.e., about 2 μm,^49,50^ or non-solvent induced phase separation (NIPS), less than 5 μm.^51^ In other cases, very large and not explicitly intended deviations in pore size were found within the same scaffold, for example, with salt leaching, ranging from 100 to 150 μm^52^ or with TIPS, even from 5 to 100 μm.^53^ A similar variability was also encountered in CNC-based scaffolds, e.g., a CNC/gelatin/bioactive glass nanocomposite scaffold, obtained by freeze-drying, had pore size variation between 50 and 250 μm,^27^ or a CNC-based hydrogel had pore dimensions varying from 20 to 500 μm.^54^

Our greater control over internal macroporosity is certainly one of the most attractive and significant advantages of using AM over traditional techniques, where the arrangement and actual pore size are not controllable variables for the fabrication of scaffolds for TE.

### 3.3 Surface Roughness

**Figure 4:**
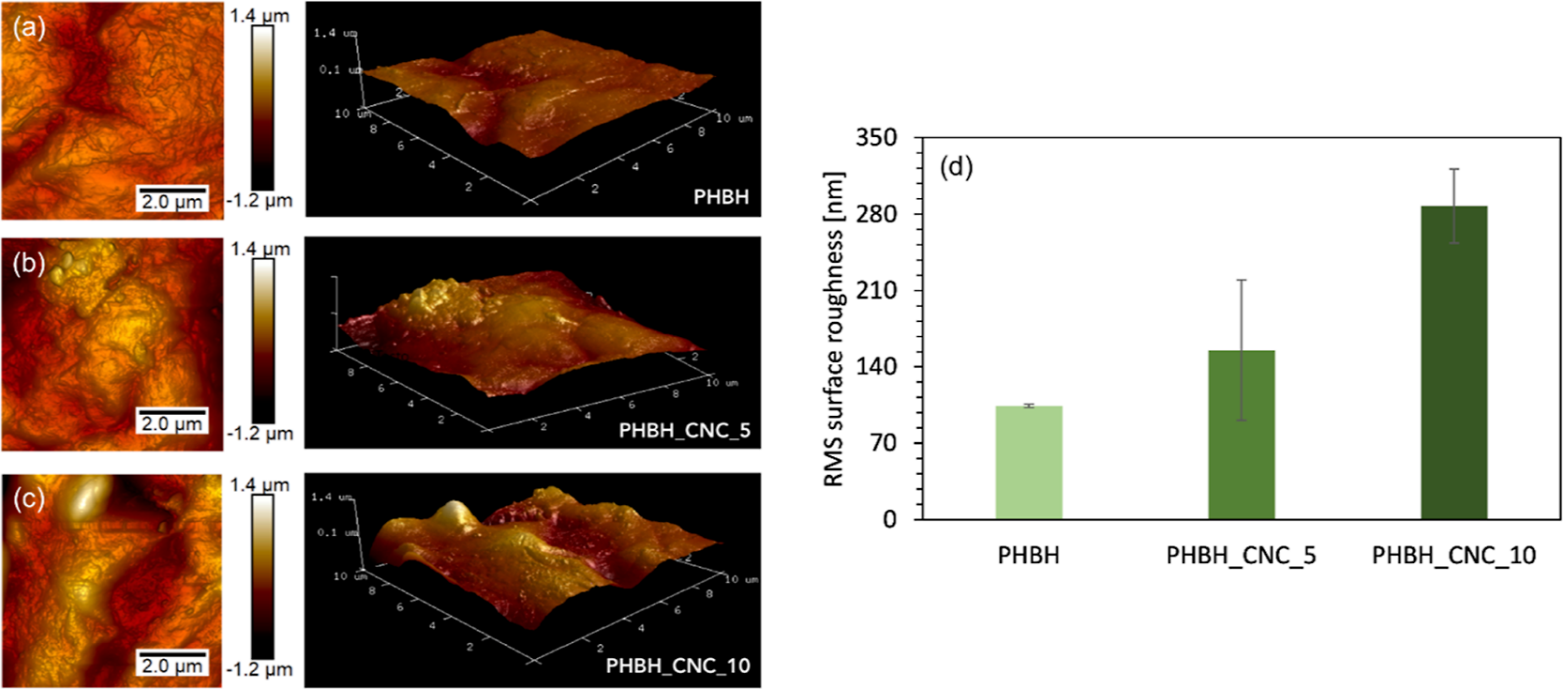
RMS surface roughness of PHBH and CNC-based biocomposites 3D-printed samples; AFM images for: (a) PHBH, (b) PHBH_CNC_5, and (c) PHBH_CNC_10. (d) Increasing RMS values as a function of CNC content. Error bars show the standard deviation for three measurements performed for each sample type on three different samples.

To study the effect of acetylated CNC content on the surface roughness of 3D-printed scaffolds, AFM measurements were carried out in tapping mode for all sample compositions on the 3D-printed plane geometries. All images of surface topographies are displayed in Figure 4 along with the RMS roughness values. The smoothest surface was obtained for neat PHBH, with an RMS value of 104 ± 2 nm. After compounding the biopolymer matrix with CNCs, the surface roughness reaches values of 155 ± 64 and 287 ± 34 nm for 5 and 10% CNC content, respectively. A similar trend was also calculated for the average roughness (R_a_), with three increasing values of 79 ± 3, 122 ± 48, and 223 ± 32 nm for the respective compositions of PHBH, PHBH_CNC_5, and PHBH_CNC_10.

Our results are in line with previous reports, where compounding CNCs with different polymer matrices, such as poly(3-hydroxybutyrate-*co*-3-hydroxyvalerate) (PHBV),^55,56^ polyvinylidene fluoride (PVDF),^57^ PLA,^58,59^ or PLA-PHB blends,^60^ led to an increase in surface roughness when the CNC content exceeded 5 wt %. The surface roughness of medical implants is a key property steering cell–biomaterial interaction, but it is highly cell type-specific.^61^ For example, Hou et al. reported the mechanosensitive response of human mesenchymal stem cells (MSCs) to surface roughness, and by varying the roughness (*R*_a_ ∼ 50–1050 nm), they showed reduced spreading area with increasing roughness but osteogenic differentiation peaking at a roughness of ∼150–280 nm,^62^ which is in the range of the here developed biocomposites. Fibroblast cells, on the other hand, showed a decreased proliferation rate with increased surface roughness (*R*_a_ ∼ 40–5070 nm),^63^ and a surface roughness value around 100 nm was suggested as optimal for fibroblast adhesion and proliferation.^64^

As in the current study, a higher CNC content corresponds with an increase in the surface roughness of the scaffolds, the PHBH scaffolds can be tuned for the final application of the 3D-printed structure, whether it is for soft or harder tissues, respectively. Besides, this study proved that compounding the PHBH biopolymer matrix with CNC is an effective way to tune the roughening morphology of biocomposites and thus improve the interaction between the cells and the scaffold to optimize the healing process.

### 3.4 Water Affinity

To establish whether the melt compounding of CNCs with the PHBH matrix could be an interesting approach to controlling the water affinity of the final biocomposites, all sample compositions were first investigated by water sorption tests and then confirmed by static water contact angle measurements. Wettability is particularly important if we consider that wettable scaffolds were demonstrated to promote human blood coagulation^65^ and, in turn, osteogenic differentiation.^66^ Besides, it is desirable for a scaffold to possess the ability to maintain a humid environment in order to promote appropriate fluid exchange between the designed part and the surrounding in vivo environment.^67^

**Figure 5:**
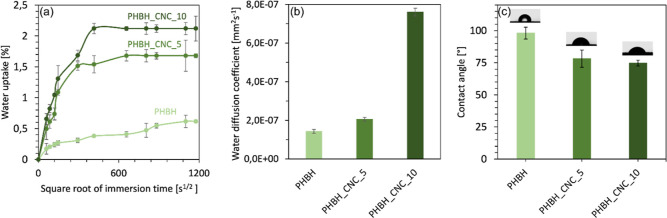
Water affinity evaluation of PHBH and CNC-based biocomposite samples; (a) water uptake versus the square root of immersion time in distilled water at 23 °C, (b) water diffusion coefficient, and (c) static water contact angle for PHBH and biocomposites at 5 and 10 wt % of CNC content.

Figure 5a shows the percentage of water absorption *M*_t_ for neat PHBH and biocomposites, with water uptake absorption increasing proportionally to immersion time until an equilibrium was achieved. All curves exhibited a typical Fickian behavior, composed of three stages: fast sorption at the initial stage, followed by a slower sorption phase, and finally presenting an asymptotic saturation. Comparing the results of neat PHBH with those of CNC-based biocomposites, we can observe a simultaneous increase in the water uptake at saturation and the saturation time, both being directly proportional to the CNC content. The water saturation time was greatly shortened from 278 h for PHBH to 117 and 44 h for biocomposites at 5 and 10 wt % CNC content, respectively. Besides, PHBH attained the minimum value of water uptake at saturation, i.e., *M*_s_ = 0.6%, compared to PHBH_CNC at 5 and 10 wt % with 1.7 and 2.2%, respectively. These differences in penetration rates of water into the material can be attributed to the different interactions that water molecules establish with the neat biopolymer matrix and CNCs, which are more hydrophilic due to the presence of acetate groups of acetylated cellulose and unreacted hydroxyl groups of cellulose.^41^ Moreover, the hydrophilic character of cellulose-based fillers involved an increase of the water diffusion coefficient, *D,* for biocomposites compared to neat biopolymers, which is consistent with previous research.^41,42^ All coefficients with the experimental standard deviation are shown in Figure 5b.

To further demonstrate the relationship between increased scaffold wettability and increased CNC content, contact angle measurements were carried out on the 3D-printed scaffolds (Figure 5c). In this case, increasing the content of CNCs decreased the static water contact angle in the biocomposites (78.2 ± 6.7 and 74.7 ± 2.3°) compared to neat PHBH (98.1 ± 4.6°), which is in good agreement with previous studies of similar biopolymer scaffolds,^68,69^ as well as for other cellulose-based composites.^27^ Together, the effects of CNC addition to PHBH on contact angle and water uptake demonstrated that by increasing the CNC content, we can increase the surface hydrophilicity of nanocomposites, which might promote cell growth within the scaffolds and thereby facilitate tissue regeneration.

### 3.5 In Vitro Degradation

**Figure 6:**
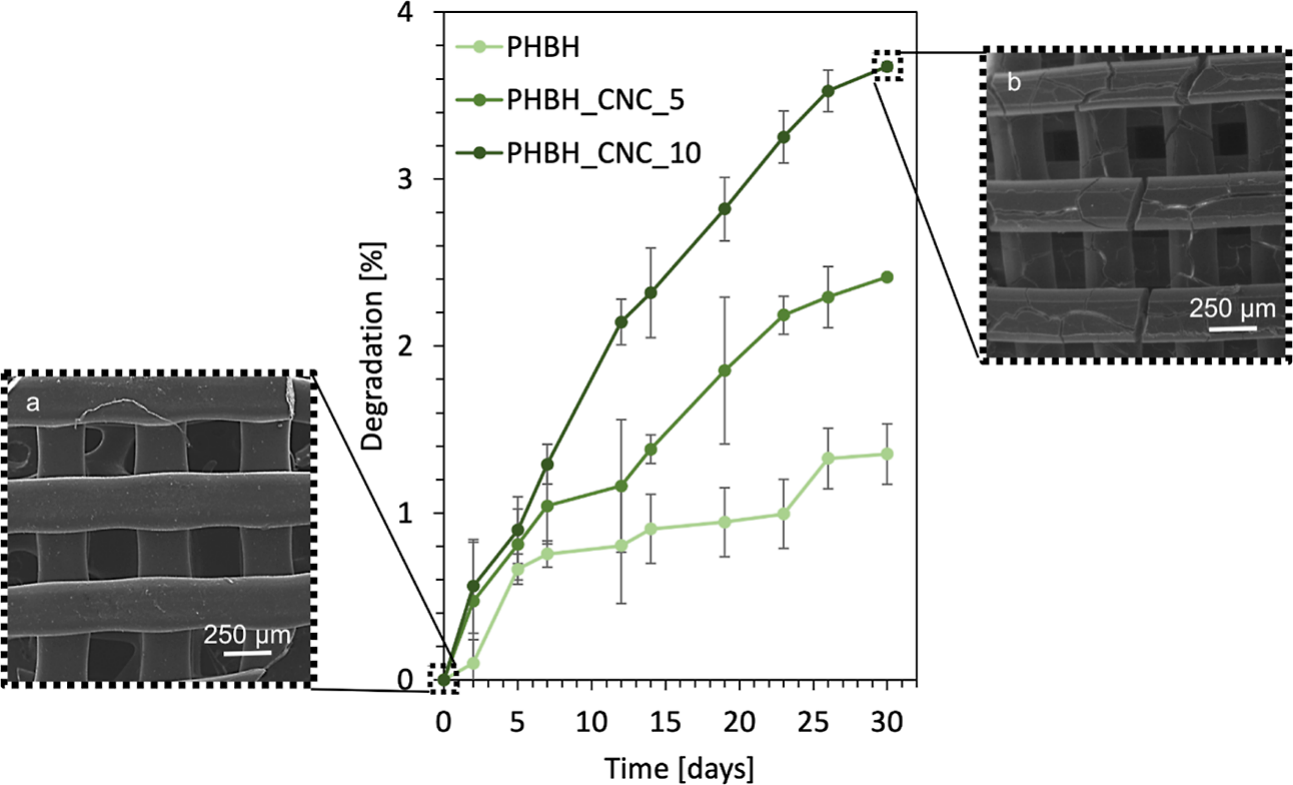
In vitro degradability of PHBH and PHBH_CNC scaffolds in 10 wt % of H_2_O_2_. All samples were submerged in 10% H_2_O_2_ and incubated for different time intervals to assess the degradation rate. Each data point represents the mean of triplicate measurements. SEM micrographs of the PHBH_CNC_10 samples at (a) the beginning and (b) after 30 days of incubation.

In vitro degradation tests were carried out for all three compositions to evaluate the effect of CNC compounding on the degradation rate of the scaffolds for up to one month following ISO standard 10993-13:2010 (Figure 6). Additionally, the morphological changes of the samples were investigated by SEM at the beginning and after 1 month of in vitro degradation. In phosphate-buffered saline (PBS) and water, almost no degradation could be observed even after 48 days of incubation (data not presented). Simulating an accelerated oxidative degradation, the weight loss for pure PHBH scaffolds was 1.3 wt %, almost doubled for PHBH_CNC_5 (2.4 wt %), and tripled for PHBH_CNC_10 (3.7 wt %) after a 1 month degradation in 10% H_2_O_2_. The effect of degradation for PHBH_CNC_10 scaffolds is particularly evident in SEM analysis, where some incipient cracks appeared (Figure 6b). This tunable degradation behavior can be attributed to PHAs undergoing a water diffusion-controlled hydrolytic degradation,^70^ and therefore, CNCs can enhance the diffusivity of the water molecules into the biocomposite matrix, which is in good agreement with the observed water affinity. Depending on the intended application of resorbable scaffolds, a faster or slower degradation process may be preferred. For example, in bone tissues, too fast degradation of the scaffold may lead to poor mechanical strength and low stiffness and thus can negatively influence bone regeneration.^71^ By varying the CNC content, it is possible to tune the final degradability of the biocomposites and thereby better tailor the scaffold properties to specific applications. Considering that several months to years are usually required for complete bone regeneration,^72^ the results suggest a possible application of PHBH–CNC biocomposites for bone tissue engineering, for which it is required to have a medium-to-long duration balance between the bone formation and scaffold resorption.

### 3.6 Cellular Attachment and Proliferation

**Figure 7:**
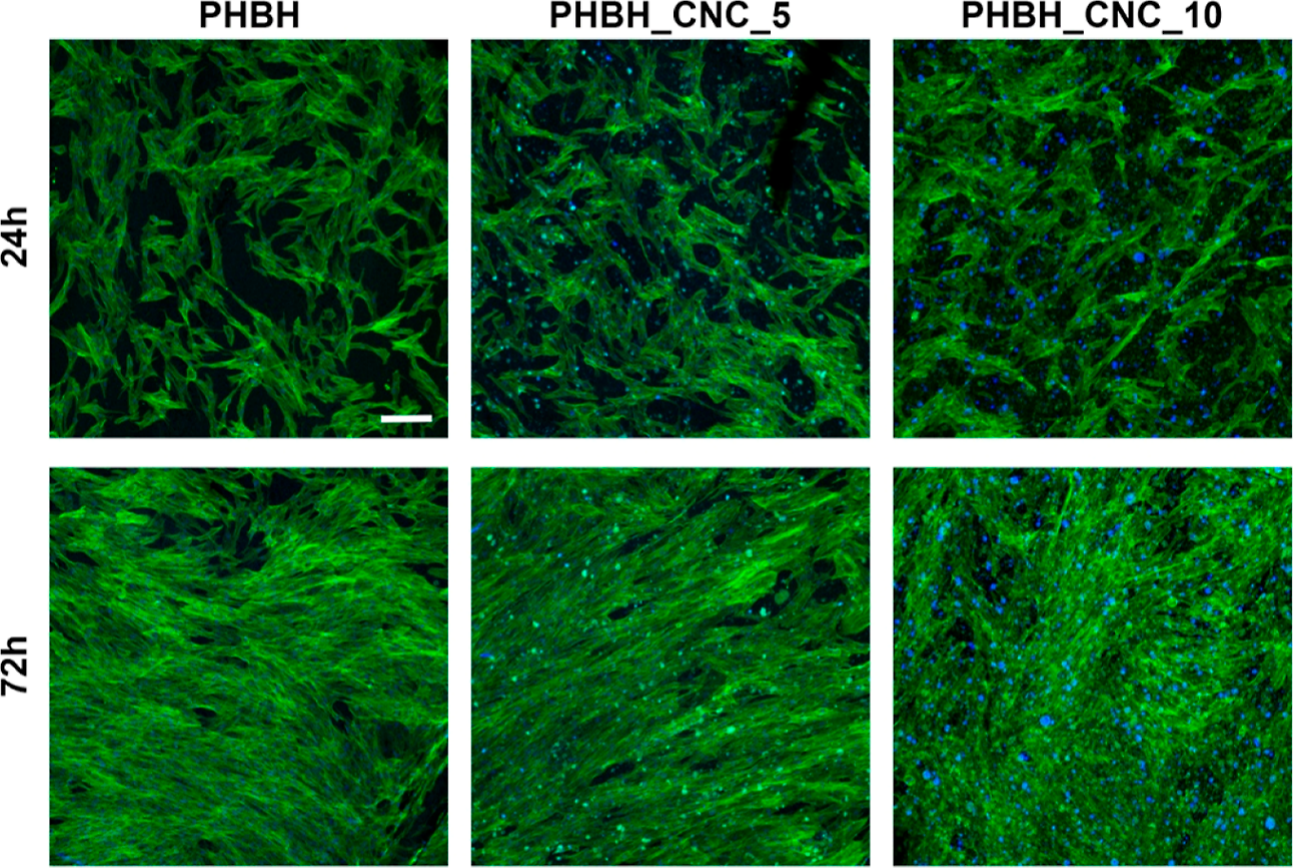
Cell attachment and proliferation. Confocal laser scanning microscopy images of NHDFs grown on PHBH and composite scaffolds for 24 and 72 h before staining for actin cytoskeleton (green) and nuclei (blue). Bright green and bright blue spots in both CNC groups represent staining artifacts. Scale bar corresponds to 200 μm.

Cytocompatibility of biocomposites was assessed by culturing NHDF for 24 and 72 h on PHBH and composite scaffolds (Figure 7). After 24 h of culture, the cells showed good attachment and a homogeneous distribution on all scaffolds, independent of CNC content. By 72 h of culture, NHDFs formed a confluent cell layer on all samples, demonstrating excellent cytocompatibility of the composite scaffolds.

These results are in line with several in vitro and in vivo studies that demonstrated the cytocompatibility of both nanocellulose^73−75^ and PHBH-based materials,^76−78^ as well as nanocellulose composites with polymer matrices other than PHBH, such as polyvinyl alcohol^79^ or PLA.^80^ This paper on the characterization of these new 3D-printed biocomposites concludes with the evaluation of the cytocompatibility of some scaffolds superficially treated with plasma, an approach commonly used to increase the surface properties of biomaterials that showed interesting bulk properties.^81^ In future developments of this work, it would be considered interesting to also assess whether and how this treatment may affect the other properties previously evaluated.

## 4 Conclusions

In this research work, we fabricated fully bio-based and resorbable scaffolds by compounding a PHBH biopolymer matrix with CNCs at 5 and 10 wt % concentrations. The biocomposites were successfully processed using the additive manufacturing approach of FFF, demonstrating a suitable 3D printability and an appropriate control over the internal macroporosities, which cannot be achieved with traditional technologies. Increasing the CNC content in the 3D scaffolds led to an increase in surface roughness, water diffusivity coefficient, and in vitro degradation rate; conversely, the static water contact angle dropped. For all compositions, the cytocompatibility of the samples was demonstrated using in vitro cell culture studies and proliferation assays. The tailoring effect of CNC therefore broadens the potential range of use of PHBH biopolymer, rendering PHBH–CNC biocomposites attractive candidates for tissue engineering applications. Further investigations and characterizations oriented toward the ability of the scaffolds to support bone formation will be required prior to consideration of in vivo studies.
